# Relationship network, knowledge transfer and open innovation behavior of SMEs from China

**DOI:** 10.1371/journal.pone.0326490

**Published:** 2025-06-18

**Authors:** Menglin Ni, Muwen Wang

**Affiliations:** School of Business Administration, Shandong Women’s University, Jinan, China; University of Southampton, MALAYSIA

## Abstract

In the context of globalization and digital transformation, small and medium-sized enterprises (SMEs) face increasing resource constraints that limit traditional innovation models. This study investigates how Chinese SMEs’ relational networks influence their open innovation behaviors, examining the mediating role of knowledge transfer and the moderating effect of innovation strategic orientation. Through questionnaire data collected from 329 SMEs’ middle and senior managers, we find that: (1) Firm relational networks significantly enhance knowledge transfer; (2) Innovation strategic orientation positively moderates the relationship between networks and knowledge transfer; and (3) Both networks and knowledge transfer exhibit strong positive impacts on SMEs’ open innovation behaviors, with knowledge transfer acting as a partial mediator. The research reveals the mechanism through which relational networks drive open innovation via knowledge mobilization while highlighting the critical regulatory role of strategic innovation focus. These findings contribute to understanding open innovation dynamics in emerging markets by demonstrating the sequential pathway: network development → knowledge acquisition → innovation outcomes. For practitioners, the study underscores the strategic value of cultivating robust relational networks aligned with innovation objectives, enhancing internal knowledge circulation capabilities, and maintaining adaptive strategic orientations to capitalize on external opportunities. The research provides theoretical and practical guidance for SMEs seeking sustainable competitive advantages through open innovation strategies in knowledge-based economies.

## 1. Introduction

The escalating complexity of the environmental landscape, economic globalization, and the data revolution have diminished the effectiveness of traditional innovation approaches and underscored the crucial role of external knowledge transfer. In recent years, the notion of open innovation has garnered considerable attention within academic circles [1–3]. The literature on open innovation behaviors has also delved into the factors that catalyze innovation, with a particular emphasis on distinguishing between conventional innovation firms and those adopting open innovation practices. Consequently, when compared to other traditional innovation models, the primary advantages of open innovation can be summarized as follows: it fosters enhanced market responsiveness, diminishes innovation costs, and adeptly addresses the challenges posed by a complex environment, blurred enterprise boundaries, and a shortened product life cycle [4,5]. Furthermore, the open innovation behavior not only amplifies the efficiency of internal research endeavors and increases the likelihood of product commercialization but also enables firms to secure competitive advantages in highly uncertain environments.

Some studies have explored the factors that influence open innovation behavior by analyzing the ramifications of specific enterprise characteristics on innovation processes, such as ownership structure, management, and internal control. Nevertheless, it is imperative to adhere to the established open innovation literature when addressing the micro-level mechanisms underpinning open innovation behaviors among SMEs, as these facets remain incompletely understood. More specifically, departing from the conventional closed innovation paradigm, these studies have scrutinized the factors that impact open innovation behavior within SMEs, encompassing intellectual capital, knowledge management capabilities, knowledge sharing practices, organizational openness and strategic orientation [6–8].

Similarly, relationship network constitutes another pivotal construct that not only enhances enterprise performance but also fosters innovative behavior [9]. Despite serving as a crucial prerequisite for open innovative behavior among SMEs, relationship network has garnered insufficient attention to at present. While numerous studies have adopted a top-down perspective, examining the work performance and motivation of individual employees [10,11]. Moreover, the purpose of our study is to analyze the impact of relationship network on the open innovation behavior of SMEs.

Despite the escalating significance of knowledge transfer, its role in strategic decisions concerning open innovation remains largely unelucidated. As an indispensable resource for innovation, knowledge embedded within social networks can effectively facilitate the dissemination of information and knowledge among enterprises, mitigate redundant investments in generic technologies, enhance innovation efficiency, and ultimately propel open innovation behavior among SMEs [12]. It is surprising that despite the pivotal role of knowledge transfer in shaping innovation decisions and behaviors, there is a scarcity of research on this topic. Notable exceptions to the conventional wisdom include the fact that knowledge transfer can actually result from heterogeneity in knowing, where diverse perspectives and expertise converge to create new insights and understandings [13]. Furthermore, the presence of innovation intermediaries plays a crucial role in fostering system-wide knowledge diffusion and growth, acting as bridges that connect disparate knowledge sources and facilitate the flow of information across organizational boundaries [14]. In recognition of this, organizations should customize their knowledge exchange practices based on their respective competitive landscapes, ensuring that these practices are tailored to their unique needs and circumstances [15]. Additionally, the importance of knowledge transfer from suppliers cannot be overstated, as it plays a vital role in improving knowledge quality and fostering innovation within organizations by bringing in fresh ideas and expertise from external sources [16]. Therefore, we undertake a comprehensive analysis to ascertain whether and how the positive and negative ramifications of knowledge transfer influence the open innovation behavior of SMEs, seeking to understand the complex interplay between knowledge transfer and open innovation strategies.

The literature on strategic management has commenced an exploration into how innovation strategic orientation influences innovation strategic decisions. However, there remains a notable dearth of research examining the strategic orientation towards innovation within SMEs and its implications for open innovation behavior related to knowledge transfer within these social networks. Firstly, innovation strategic orientation assumes a pivotal role in SMEs, where economic and social relationships intertwine. Secondly, the significance of strategic cognitive and behavioral innovation becomes particularly pronounced when tasks are complex and technical knowledge is challenging to acquire within a constrained timeframe. Given that each strategic decision can profoundly impact the performance of SMEs, and managing open innovation is inherently complex and difficult to control, analyzing the effects of innovation strategic orientation on knowledge transfer management offers a comprehensive understanding of the unique dynamics underlying the open innovation behavior of SMEs.

However, despite this recognition, there remains a research gap concerning the nuanced mechanisms concerning the specific mechanisms underlying the open innovation behavior of SMEs influenced by their relational networks, particularly the mediating role of knowledge transfer and the moderating role of innovation strategy orientation. Existing research often discusses the direct impact of relational networks on knowledge acquisition or innovation performance in isolation, yet seldom comprehensively considers the bridging role of knowledge transfer between relational networks and open innovation behavior, as well as how innovation strategy orientation functions as a moderating variable influencing the effectiveness of this process. Therefore, this study focuses on uncovering the complex interaction mechanisms among relational networks, knowledge transfer, innovation strategy orientation, and the open innovation behavior of SMEs, providing theoretical and practical guidance for enterprises to effectively promote open innovation to achieve their strategic objectives, thereby deepening the understanding of how SMEs can leverage their relational network advantages to achieve sustainable development through open innovation strategies in the context of global competition. Drawing on survey data from 329 SMEs in China, the research delves into the mediation of knowledge transfer in the relationship between relationship network and open innovation behavior, as well as the moderating effect of innovation strategy orientation on this relationship. The identified research void underscores the need for a more comprehensive understanding of how and under what conditions relationship network can effectively promote open innovation among SMEs, thereby providing actionable insights for firms seeking to leverage their networks to achieve strategic innovation objectives.

The study of the mechanism underlying open innovation behavior in SMEs, particularly within the context of China, presents a unique and significant backdrop for several compelling reasons. China, being the largest developing economy and a pivotal player in global commerce, offers a fertile ground for exploring the nuances of SME innovation strategies influenced by intricate relationship network. The selected background of Chinese SMEs is particularly relevant due to the country’s distinct economic landscape, characterized by rapid technological advancements, robust government policies promoting innovation, and a deeply embedded cultural emphasis on networking and social capital. This unique setting provides an ideal environment to investigate how corporate relationship network impacts open innovation behavior, given that SMEs in China often rely heavily on their networks for resource acquisition, market access, and knowledge exchange. Furthermore, the rapid evolution of innovation ecosystems in China necessitates an understanding of how firms navigate these complex networks, facilitated by knowledge transfer and shaped by their innovation strategy orientations. Furthermore, as China continues to undergo rapid economic transformation and technological advancements, the role of innovation strategy orientation in moderating the relationship between relationship network and knowledge transfer becomes even more crucial. Therefore, the selected background of Chinese SMEs offers a rich and nuanced setting for exploring the complex mechanics of relationship network on open innovation behavior, yielding insights that are not only relevant to the Chinese context but also contribute to the broader understanding of SME innovation strategies globally.

Considering the importance of the corporate social network to open innovating behavior of SMEs in China, the influence mechanism of knowledge transfer and innovation strategic orientation need explore further. This study of the influence of corporate relationship network on the open innovation behavior of SMEs is particularly pertinent and unique when examined within the context of China. The SME sector constitutes a vital component of its economy, driving innovation, job creation, and economic growth in China. This backdrop is significant because the SMEs often operate in a highly competitive and resource-constrained environment, necessitating innovative strategies to survive and thrive. The unique aspect of the Chinese context lies in the prevalence of strong relational ties and the extensive use of personal networks for business purposes, which is deeply embedded in Chinese culture and business practices. This cultural emphasis on networking provides SMEs with a distinct advantage in accessing resources, sharing knowledge, and collaborating with other firms. By leveraging survey data from 329 SMEs in China, this paper aims to elucidate the intricate interplay between relationship networks, knowledge transfer, and open innovation behavior, highlighting the moderating role of innovation strategy orientation. Such an investigation not only contributes to the theoretical advancement of open innovation research but also offers practical insights tailored to the specific challenges and opportunities faced by Chinese SMEs, guiding them on effective strategies to leverage their networks for fostering open innovation and achieving strategic objectives.

Therefore, the main contributions of this paper list as follows:

(1) Theoretical Integration of Social Capital and Open Innovation: This study advances relationship network theory by repositioning it as a critical social capital mechanism that drives open innovation behavior in SMEs. Unlike prior literature confined to individual leadership or dyadic business ties, it empirically examines the structural, procedural, and governance dimensions of open innovation systems, thereby broadening the theoretical boundaries of both relationship network and open innovation paradigms.
(2) Mediating Mechanism of Knowledge Transfer: By conceptualizing and validating knowledge transfer as a pivotal mediator, the research elucidates how relational networks indirectly shape SMEs’ open innovation practices. This integrative framework addresses a critical gap in existing scholarship, which has fragmented the nexus among networks, knowledge dynamics, and innovation outcomes, thereby offering a nuanced understanding of value creation pathways in open innovation ecosystems.
(3) Strategic Orientation as a Contextual Moderator: The study contributes to strategic management literature by identifying innovation strategic orientation as a boundary condition that modulates the efficacy of relational networks on knowledge transfer. This finding challenges dominant perspectives that overlook strategic heterogeneity, instead demonstrating that proactive versus reactive innovation postures differentially amplify or constrain the knowledge mobilization potential of SMEs’relational capital.

This paper has been organized as follows: Section 2 synthesizes literature to establish the theoretical framework, linking relationship network, open innovation, and knowledge transfer while contextualizing innovation strategic orientation. Section 3 details the methodology, outlining sample criteria, construct measurement, and structural equation modeling. Section 4 reports empirical results, testing mediation and moderation effects. Section 5 discusses theoretical and practical implications, embedding findings within SME innovation resilience debates. Section 6 concludes by summarizing contributions, acknowledging limitations, and proposing future research directions.

## 2. Literature analysis and research hypothesis

### 2.1. Relationship network and knowledge transfer within enterprises

After decades of development, relationship networks have emerged as a significant topic in management research, resulting in notable conclusions [17,18]. Research has demonstrated that relationship capital, as a strategic resource for enterprises, is embedded within the relationship network and has a positive impact on corporate governance, enterprise growth, technological innovation, production efficiency, financial performance, knowledge sharing and mobility [19,20]. The corporate relationship network, as the primary conduit for social capital, can be categorized into two distinct forms: the internal relationship network, which encompasses the relationships among employees of the enterprise, and the external relationship network, which refers to the relationships that an enterprise has with organizations external to the enterprise itself [21].

Knowledge transfer between enterprises has been recognized as the most significant resource for the maintenance and transmission of corporate relationship networks. This process encompasses knowledge exploration and absorption, knowledge connection and retention, and knowledge deconstruction and utilization [22]. In essence, Knowledge Management can be defined as the process of capturing and transforming internal and external knowledge resources within an organization, including how knowledge flows. From the perspective of internal relationship networks, common goals can facilitate knowledge exchange among employees within an enterprise, thereby promoting the transformation of knowledge [23]. In essence, a robust internal relationship network can reduce the cost of internal knowledge transfer by enhancing the effectiveness of knowledge exchange. It is evident that the frequency of communication within the network directly correlates with the extent of knowledge transfer [24]. Conversely, the relationship network external to the enterprise facilitates knowledge transfer within the enterprise by enhancing the scale, centrality, or connectivity of the network. The rationale behind this phenomenon is that the number of channels for knowledge transfer is directly proportional to the size of an enterprise’s external relationship network [25]. The efficiency of knowledge transfer is directly impacted by the number and frequency of channels, which is reflected in the external relationship network center. The greater the external relationship network degree, the higher the business center in the external relationship network degree is, and the faster the knowledge transfer is [26]. This indicates that enterprises with greater absorptive capacity can effectively utilize the knowledge transferred. The external relationship networks of enterprises have been shown to provide increased opportunities for the acquisition of information, knowledge, and resources [27]. Corporate relationship networks have been shown to enhance both the original social relations of enterprises and the relations among organizational employees, thereby affecting knowledge transfer [28]. Previous models examining the factors influencing open innovation behavior in SMEs have often overlooked the nuanced roles played by relationship networks, innovation strategy orientation, and knowledge transfer. Most models typically focus on internal capabilities and resource availability within SMEs, neglecting the external dynamics that significantly shape their innovation strategies. For instance, many prior studies have underestimated the extent to which SMEs leverage their relationship networks to facilitate knowledge transfer, a crucial process for fostering open innovation. Therefore, studying the impact of corporate relationship networks on knowledge transfer is of great significance.

H1: Relationship network has a positive impact on knowledge transfer between enterprises.

### 2.2. Analysis of the moderating effect of innovation strategy orientation on relationship networks and open innovation behavior of SMEs

The innovation strategy orientation enhances the positive impact of relationship networks on knowledge transfer, because innovation strategy orientation can be interpreted as an understanding that when enterprises face internal and external environmental challenges and resource constraints, in order to reduce R&D costs and cycles, enterprises will efficiently promote relationship networks to integrate resource channels external to the enterprise for updating internal knowledge reserves, thus facilitating knowledge transfer [23]. It is evident that the stronger the innovation strategy orientation, the greater the need for enterprises to engage in timely knowledge and information sharing to maintain advanced and unique production and R&D activities. This heightened focus on relationship networks as a conduit for knowledge transfer results in the accumulation of a substantial knowledge reserve, thereby accelerating the open innovation behavior of SMEs [29]. Conversely, the innovation strategic orientation exerts a significant influence on enterprises, compelling them to meticulously cultivate their own relationship networks to further enhance the efficiency of knowledge transfer through high-intensity corporate communication and sectoral coordination [30]. Meanwhile, The relationship between innovation strategic orientation and knowledge transfer is strengthened by the strength of the relationship networks, which in turn increases knowledge reliance and credit commitment [31]. Moreover, these models have generally failed to account for the moderating role of innovation strategy orientation, which can amplify or dampen the effectiveness of relationship networks in facilitating knowledge exchange. By recognizing the importance of a strong innovation strategy orientation, SMEs can better navigate their relationship networks to extract and apply valuable knowledge, thereby enhancing their open innovation capabilities. In summary, companies with a stronger innovation strategy orientation pay more attention to the role of relationship networks in knowledge transfer. They pursue and obtain new opportunities by investing in corporate networks to improve the efficiency of inter-enterprise knowledge transfer. Therefore, we propose the following hypothesis.

H2: Innovation strategy orientation will strengthen the positive influence of relationship networks on knowledge transfer.

### 2.3. Analysis of the moderating effect of innovation strategic orientation on relationship network and enterprise open innovation behavior of SMEs

Compared with traditional innovation, open innovation is understood as an innovation activity that is driven by exchange and integration of external elements, including the exchange and transformation of knowledge, technology, capital, manpower and ideas [32]. Through cooperation with external enterprise organizations, external knowledge can be absorbed by sharing or introducing, which can be developed and utilized scientifically, so as to maintain the competitive advantage [33]. In recent years, research on SMEs has found that a strong corporate relationship network can positively impact enterprise innovation activities, including the development of new products, breakthroughs in new technologies, and the generation of new ideas [34]. From a knowledge perspective, SMEs engage in open innovation behavior driven by knowledge transformation. It is insufficient to rely solely on internal knowledge, new knowledge transferred from external sources plays a crucial role [35]. The advent of the Internet has enhanced the efficiency of knowledge transfer and concomitantly led to a reduction in the associated costs, thereby encouraging enterprises to prioritize open innovation activities as a means of enhancing their competitive prowess through innovation.

In addition to relationship networking, knowledge transfer is a key driver of enterprise open innovation activity. Firstly, within the constraints of limited resources, the enterprise relationship network serves as the foundation for employees to learn and reference across the organization [36]. Through knowledge transfer, enterprises can accumulate a substantial body of novel knowledge. The enterprise must then integrate and allocate these resources effectively and reasonably, thus greatly increasing the probability of new thinking and ideas [37]. The relationship network has been demonstrated to have a significant impact on the open innovation behavior of SMEs by facilitating knowledge transfer between enterprises [38]. In the context of open innovation research, numerous scholars have underscored the significance of relationship networks in influencing the open innovation behavior of SMEs through [39]. Additionally, previous models examining the factors influencing SMEs have often overlooked the nuanced roles played by relationship networks and knowledge transfer in fostering open innovation behavior. These models typically focus on individual firm-level attributes such as resources, capabilities, and technological advancements, neglecting the interconnections and collaborative dynamics within and across industries. Furthermore, previous research has rarely explored the mediation effect of knowledge transfer in the relationship between networks and open innovation behavior, which is crucial for elucidating the mechanisms through which networks influence SMEs’ innovation strategies. This mediation effect underscores the importance of facilitating knowledge sharing and integration within SME networks to stimulate innovative practices. Thus, by incorporating the dimensions of relationship networks and knowledge transfer, and by examining their interconnected roles in influencing open innovation behavior, our hypotheses provide a more robust framework for understanding the innovation dynamics of SMEs. This nuanced perspective not only strengthens the theoretical foundation but also offers practical insights for policymakers and SME managers seeking to enhance their innovation capabilities through strategic networking and knowledge management practices. The subsequent hypotheses are proposed in this paper.

H3: Relationship network has a positive impact on open innovation behavior of SMEs of enterprise.

H4: Knowledge transfer positively impacts the open innovation behavior of SMEs.

H5: Knowledge transfer mediates the relationship between the network and the open innovation behavior of SMEs, the influence of the network on the open innovation behavior of SMEs is strengthened by knowledge transfer.

**Fig 1:**
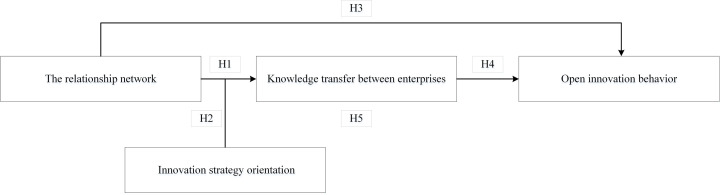
Hypothetical model.

Based on the literature review and theoretical assumptions deduced above, this paper concludes the representative papers summarized in Table 1, and builds a moderated mediation model as shown in Fig 1.

**Table 1:** Representative literature summary.

Study Area	Advantages	Limitations	Reference
The comprehensive relationship between knowledge spillover and corporate innovation behavior.	Introducing the dynamic moderating effect of psychological trust into the analysis of the relationship between knowledge spillover and open innovation.	The limitation of the sample to Chinese high-tech enterprises restricts the applicability of the conclusions to other cultural contexts.	[40]
The impact of cross-border knowledge transfer on the innovation performance of SMEs in developing countries.	This study emphasizes that small and medium-sized enterprises (SMEs) should strengthen domestic collaborations to enhance the efficiency of cross-border knowledge transformation, aligning in global supply chains.	The failure to distinguish industry differences within the sample restricts the applicability of the conclusions to heterogeneous industries.	[41]
Assessment of organizational attitudes towards open innovation and organizational open innovation capability.	Based on a mixed sample of MNCs and local enterprises, with a focus on Open Innovation practices in the South Korean market, the study reveals the role differentiation and strategic orientation differences of various organizations within the innovation ecosystem.	The failure to differentiate between industries or scale variations among multinational enterprises in the sample restricts the applicability of the conclusions.	[42]
Correlation model between social network theory and knowledge-based view.	By linking the dynamic evolution of firms’ internal collaboration networks with knowledge search behaviors, the research uncovers a nonlinear pattern of optimal moderate dynamism.	The restriction of the sample to China’s C39 industry, which does not encompass service-oriented or low-technology-intensive industries, may render the conclusions inapplicable to firms relying on non-technical knowledge.	[43]
Focusing on the interactive relationship between knowledge transfer mechanisms and the decentralization within franchise networks.	Knowledge-mediator-decentralization fit model is proposed, offering a new interpretation of knowledge flow perspectives within network governance theory.	The limitation of the sample to European countries or regions, without including franchise networks in high power distance cultures, may underestimate the moderating effect of cultural norms on the acceptance of decentralization.	[44]
The Relationship between Strategic Orientation, Networking Capability, Networking Ability, and New Product Development Performance	This research unveils the key pathways for enhancing innovation performance among firms in emerging markets, providing crucial theoretical support for strategic resource allocation.	The restriction of the sample to three industries in China, which does not cover high-technology or service-intensive industries, may limit the conclusions to specific industry attributes.	[45]

## 3. Research design

### 3.1. Sample and data collection

This paper tests the hypotheses proposed above by collecting data from a questionnaire survey, and the respondents are mainly middle and senior managers who have good understanding of the enterprise’s innovation decisions and operating conditions. These managers occupy pivotal roles in coordinating cross-functional collaboration, external partner engagement, and resource allocation—activities that are central to shaping relationship network dynamics and facilitating open innovation implementation, as evidenced by prior research. Their positional authority enables access to holistic organizational data, including proprietary knowledge flows and strategic innovation decisions, which lower-level employees may not fully observe. Furthermore, middle managers act as critical intermediaries in translating top-level strategic orientations into operational practices, thereby bridging macro-level network strategies and micro-level innovation behaviors [46]. The demographic details of the respondents are summarized in Table 2. The data collection process has been divided into two stages. The first stage is a pre-survey, in which 100 questionnaires are issued and modified and improved according to the questions fed back by the respondents during the survey. This iterative process was instrumental in ensuring the clarity, relevance, and absence of ambiguity in the questions, thereby mitigating the risk of potential common method bias and systematic errors.

**Table 2:** Demographic Characteristics of the Sample.

Variables	Options	Sample size	Rate
Gender	Male	197	59.88%
	Female	132	40.12%
Age	30 and below	25	7.60%
	30-39	82	24.92%
	40-49	135	41.03%
	50-59	83	25.23%
	60 and above	4	1.22%
Education Level	Middle school and below	0	0.00%
	High school	9	2.74%
	Junior college	34	10.33%
	Bachlor	182	55.32%
	Master and above	104	31.61%

The second stage is a formal survey. To ensure methodological rigor, the survey has been administered electronically to mitigate geographical constraints and facilitate efficient data aggregation. Specifically, a structured questionnaire was distributed online via Wenjuanxing platform, a professional survey platform widely adopted in Chinese management research [47]. Potential respondents were identified through corporate directories and industry associations, with invitation emails containing anonymized survey links dispatched to middle and senior managers across 329 SMEs located in Shandong, Shanghai, Beijing, and other provinces or cities in China. This geographical distribution was strategically designed to capture regional economic diversity while aligning with China’s SME concentration patterns. 386 questionnaires were collected in this stage, and 329 valid questionnaires remained after removing some unqualified ones, which diversified the respondent pool and minimized the impact of any localized biases. Additionally, the authors rigorously screened the collected questionnaires, resulting in 329 valid responses from SMEs after excluding unqualified ones. This careful selection process further enhanced the reliability and generalizability of the data. These regions were chosen based on their varying economic development levels, industrial structures, and innovation ecosystems. Shandong, as an economically vibrant province with a diverse range of industries, provides insights into the innovation practices of enterprises in a robust industrial base. Shanghai, known for its advanced technological development and international business environment, offers a perspective from a highly urbanized and innovative metropolis. Beijing, being the capital of China, houses numerous headquarters of large enterprises and research institutions, reflecting the policy-driven innovation landscape. Together with other provinces and cities included in the survey, these regions help to mitigate potential sample representative issues by encompassing a broad spectrum of enterprise types, sizes, and innovation capabilities. By collecting data from respondents across these diverse geographical areas, the study aims to achieve a more comprehensive understanding of enterprise innovation decisions and operating conditions in China. Finally, the recruitment period spanning from October 9, 2024, to November 10, 2024, allowed ample time for respondents to provide thoughtful and considered responses, reducing the risk of rushed or cursory answers to minimizing common method bias in their data collection process.

In this study, participant consent constituted a pivotal aspect of the ethical considerations undertaken. Prior to their involvement in the research, it was imperative to ensure that all participants had been furnished with a comprehensive explanation of the study’s intricacies, and had subsequently provided their consent. Specifically, within the survey questionnaire itself, a detailed ethics statement was included, clearly outlining the purpose of the study, the nature of their participation, how their data would be used, and measures taken to ensure their anonymity and confidentiality. Participants were explicitly informed that their involvement was voluntary and that they had the right to withdraw at any time without any consequences.

The consent obtained was of the written variety. Prior to commencing the survey, participants were required to read through the ethics statement and affirmatively indicate their consent by selecting a checkbox or similar digital option before they could proceed with completing the questionnaire. This method ensured that participants were fully aware of the study details and had given their informed consent. It also provided a clear and documented record of their agreement, in accordance with ethical guidelines.

### 3.2. Measurement of variables

The variables were measured on a Likert scale, with the exception of control variables such as such as the year of establishment of the enterprise, the number of employees, the nature of the enterprise, the industry in which the respondents worked and their positions of the respondents. Details are as follows:

(1) Relationship network. Referring to the scale of West & Bogers [48], 8 items are measured, including “①In general, the relationship between department members is good. ②The individuals in my department consistently demonstrate honesty and are reliable. ③Those in my department display a high level of ethical behavior. ④I have complete confidence in the members of my department. ⑤There is an atmosphere of cooperation and trust in The company’s agreements with other companies to develop new products and improve existing products. ⑥The firms involved in our collaborative pacts show a strong level of dedication towards our undertakings. ⑦The firms in our collaborative agreements are aligned in their objectives and interests regarding our joint endeavors. ⑧The firms under our collaborative agreements hold a shared perspective on the ecosystem and crucial elements for success.”
(2) Knowledge transfer. Referring to the scale of Cuevas-Rodriguez et al. [49], measurement is carried out through four items, representing as “①If I acquire new knowledge externally, I actively share it by presenting at internal workshops or seminars. ②Due to the cooperation with related enterprises, we have acquired a lot of management skills and knowledge. ③If partners pose questions to me, I actively share my knowledge by providing answers. ④We have improved the design of our products by cooperating with related companies.”
(3) Open innovation behavior of SMEs. Referring to the paper of Lichtenthaler [50], the measurement is carried out through 9 items, representing items such as “①The company frequently analyzes the external environment and incorporates external technologies, information, concepts, knowledge, etc. ②The company actively seeks external knowledge and technology sources during the process of developing new products or technologies. ③The company believes that leveraging external knowledge and technology sources serves as a beneficial supplement to the company’s R&D efforts. ④The company often integrates externally developed knowledge and technologies into the company’s R&D activities. ⑤The company searches for technologies and patents from other enterprises, R&D institutions, or universities, and actively explores avenues for commercialization by external organizations. ⑥The company believes that commercialization by external organizations is conducive to enhancing the company’s R&D performance. ⑦The company transfers R&D projects that have not been completed or have been terminated midway to third-party organizations. ⑧The company participates in the commercialization activities of new technologies or new product projects of other enterprises, with the aim of expanding market share or developing new markets. ⑨The company occasionally sells intellectual property rights to obtain commercial value.”
(4) Innovative strategic orientation. Referring to the study of Huang [51], measurement is carried out through three items, representing items such as “①The company frequently engages in the development of new products or new technologies. ②The company invests substantial capital in the innovation of production processes or service delivery processes. ③The company often takes the lead in introducing new products or new services to the market.”

## 4. Data analysis

### 4.1. Reliability and validity test

This study uses the mature Cronbach’s α coefficient to test the reliability of independent variables such as relationship network, knowledge transfer, open innovation behavior of SMEs and innovation strategic orientation. Concurrently, the KMO value and the Bartlett spherical value, as reported by the orthogonal variance maximization rotation method and the principal component method, were utilized to assess the validity. As demonstrated in Table 3, the Cronbach’s α values for all variables exceed 0.9, signifying that the relationship network, knowledge transfer, open innovation behavior of SMEs scale, and innovation strategy orientation scale demonstrate excellent reliability. In conclusion, the results collected by questionnaires are highly reliable and can be used to verify the hypothesis of this study. Furthermore, the KMO values of each variable were 0.908, 0.849, 0.918 and 0.756, respectively, all greater than 0.7, and the significance probability of the Bartlett sphericity test value was 0, less than 0.01, with principal component factor loadings all greater than 0.7, indicating that the structure validity of each scale was good. The scale effectively reflected the real level of relationship network, knowledge transfer, open innovation behavior of SMEs and innovation strategic orientation of the respondents.

**Table 3:** Reliability and validity test of all variable measurement scales.

Variable	α	KMO	Bartlett test	item	factor	item	factor	item	factor
The relationship network	0.949	0.908	P = 0.000	PI-1	0.827	PI −2	0.883	PI −3	0.873
				PI −4	0.871	PI −5	0.850	PI −6	0.845
				PI −7	0.862	PI −8	0.859		
Knowledge transfer	0.949	0.849	P = 0.000	PI-12	0.919	PI −13	0.925	PI −14	0.951
				PI −15	0.931				
Open innovation behavior of SMEs	0.944	0.918	P = 0.000	PI-16	0.841	PI −17	0.865	PI −18	0.850
				PI −19	0.870	PI −20	0.843	PI −21	0.864
				PI −22	0.834	PI −23	0.714	PI −24	0.804
Innovation strategy orientation	0.906	0.756	P = 0.000	PI-9	0.914	PI −10	0.920	PI −11	0.919

### 4.2. Descriptive analysis

Table 4 shows the mean, standard deviation and matrix of correlation coefficients for each of the research variables. The relationship network of the respondents (3.9328), innovation strategic orientation (3.1905), knowledge transfer (3.2409) and open innovation behavior of SMEs (3.3131) are all above the medium level compared to the median of 3 (five points). In line with the theoretical premise of the paper, the surveyed companies have established a relationship network to some extent and recognize the importance and necessity of open innovation behavior for SMEs. Furthermore, regarding the correlation analysis, firstly, relationship network, open innovation behavior of SMEs, innovation strategic orientation and knowledge transfer are significantly positively correlated (correlation coefficients are all > 0, P values are all < 0.01). The correlation coefficients between relationship network and other variables are knowledge transfer (0.536), open innovation (0.491) and innovation strategic orientation (0.456), which indicates that relationship network has a moderate positive correlation with open innovation behavior of SMEs and knowledge transfer, which provisionally supports hypotheses H1 and H2. Second, the positive correlation coefficient between knowledge transfer and open innovation reaches 0.711, which provisionally supports hypothesis H3. Compared with the relationship network, the correlation coefficient between knowledge transfer and open innovation behavior of SMEs is higher and shows a closer correlation, which indicates that knowledge transfer may act as a mediator in the relationship between network and open innovation behavior of SMEs. Therefore, hypothesis H5 is provisionally supported, which provides a basis for testing the mediating effect. In addition, the positive correlation value between innovation strategic orientation and knowledge transfer is 0.723 (P < 0.01), which preliminarily indicates that there may be a moderating relationship between innovation strategic orientation and knowledge transfer. It is hypothesized that further investigation of H4 is required in subsequent studies. Moreover, prior to the regression analysis, a variance inflation factor (VIF) diagnosis was conducted for all variables entered into the regression model. The outcomes of this investigation revealed that the VIF values for all variables were less than 2.5, which is well below the 10 thresholds typically considered to indicate a lack of multicollinearity. These findings provide a solid research foundation for further exploring the influence mechanism between relationship network and open innovation behavior of SMEs.

**Table 4:** Mean values, standard deviations and correlation coefficients of variables.

variable	mean	Standard deviation	Relationship network	Innovation strategy orientation	Knowledge transfer
Relationship network	3.93	0.7609			
Innovation strategy orientation	3.19	1.0048	0.456^**^		
Knowledge transfer	3.24	0.9437	0.536^**^	0.723^**^	
Open innovation behavior of SMEs	3.31	0.8426	0.491^**^	0.667^**^	0.711^**^

** Significantly correlated at 0.01 level.

### 4.3. Hypothesis testing analysis

The hierarchical multiple regression analysis for testing the mediation and moderation effects is applied in this study due to it allows for a clear and systematic examination of the relationships among the predictor variables, mediator, and dependent variable that make it particularly suitable for the current investigation. By entering variables in a specified order, researchers can isolate the unique contributions of each variable to the model, providing insight into the mechanisms through which relationships operate. This is particularly crucial for testing mediation hypotheses, as it enables the assessment of whether the effect of the predictor on the dependent variable is partially or fully explained by the mediator. Furthermore, hierarchical multiple regression allows for a step-by-step examination of the research model, enabling the authors to isolate the unique contributions of each predictor and mediator. This sequential analysis enhances the understanding of how different components of the model interact and contribute to the outcome, which is crucial for generating actionable insights for SMEs managers and policymakers. In summary, the choice of hierarchical multiple regression in this study was driven by its clarity in testing mediation and moderation and ability to provide sequential analysis of the research model, all of which strengthen the argument and validity of the findings. SPSS25.0 was used in this paper to test the hypothesis mentioned above through hierarchical multiple regression, and the results are shown in Table 5.

**Table 5:** Multiple regression results.

variable	Knowledge transfer		Open innovation behavior of SMEs		
	M1	M2	M3	M4	M5
**Control variables**					
Years of working	−0.0982	−0.0197	−0.0978	−0.0422	−0.0447
position	−0.0550	−0.0756	−0.0637	−0.0360	−0.0340
Set up the year	0.0168	0.0133	0.0056	−0.0051	−0.0034
The number of employees	0.0746	−0.0337	0.0812	0.0326	0.0409
Enterprise nature	−0.0178	0.0435	−0.1394	−0.1442	−0.1298
industry	−0.0830	−0.0460	0.0232	0.0736	−0.0680
**Explanatory variables**					
The relationship network	0.6592^***^	0.0121	0.5295^***^		0.1736^***^
Knowledge transfer				0.6159^***^	0.5398^***^
**Intervening variable**					
Innovation strategy orientation		0.1898			
Relationship networking ^*^ Innovative strategic orientation		0.0958^*^			
F	21.38^***^	50.65^***^	19.03^***^	50.77^***^	47.44^***^
R^2^	0.318	0.588	0.293	0.525	0.543

Note: n = 306;

*** represents P < 0.01,

** represents P < 0.05,

* represents P < 0.1; Constant terms are not reported in this table.

The control variables employed for the verification of the hypothesis via hierarchical regression analysis included working years, position, establishment year, number of employees, enterprise nature and industry. The following models are included in the hierarchical regression analysis: Model M1 is employed for regression analysis of relationship network, while Model M2 is used for regression analysis of knowledge transfer and open innovation behavior of SMEs. The third model, M3 is utilized for regression analysis of the relationship network, knowledge transfer, and open innovation behavior of SMEs. The mediating effect of knowledge transfer is further tested. The fourth model, M4, is employed to regress the network of relationships and transfer knowledge. Model M5 focuses on the relationship network and innovation strategy-oriented product items, utilizing regression analysis to examine knowledge transfer. The regression coefficient and model are tested for significance, and the results are used to explain and control variables in the standardized processing of the product item. The central relationship between the firm’s network and the regulation of knowledge transfer is a key aspect of the innovation strategy. As demonstrated in Table 5, the regression results are evident.

(1) In model M1, the relationship network is used as the explanatory variable and the transfer of knowledge is used as the explained variable. The F-value of model M1 is 21.38 and the coefficient is 0.6592, both passing the significance test, indicating that corporate relationship network significantly positively affects knowledge transfer. Therefore, H1 can be verified.
(2) According to the regression results of model M2, when the normalized product term of relationship network and innovation strategy orientation is added into the model, the regression coefficient of the product term is 0.0958 and has significance (P < 0.1), R2 = 0.588, F value is 50.65, and significance probability P = 0.000. H2 demonstrates that an innovation strategic orientation has a positive moderating effect on the relationship between an enterprise’s relationship network and knowledge transfer. In other words, the stronger the innovation strategic orientation, the greater the positive impact of the relationship network on knowledge transfer.
(3) In model M3, relationship network is taken as the explanatory variable, and open innovation behavior of SMEs is the regression model of the explained variable. M1 results show that R2 = 0.293, the model F value is 19.03, and the significance test is passed. The regression coefficient of relationship network of explanatory variable is 0.5295, which is significant at 1% level. This indicates that enterprise relationship network has a positive impact on open innovation behavior of SMEs, which is verified by hypothesis H3.
(4) In model M4, the regression coefficient of knowledge transfer on open innovation behavior of SMEs is 0.6159, which passes significance test (R2 = 0.525, F value is 50.77, significance probability P = 0.000), indicating that knowledge transfer has a significant positive impact on open innovation behavior of SMEs of enterprises, which is proved by H4.
(5) In model M5, the regression coefficients of relationship network and knowledge transfer on open innovation behavior of SMEs of enterprise are 0.1736 and 0.5398 respectively, R2 = 0.543 and F value is 47.44, both of which pass the significance test. M5 added knowledge transfer variable on the basis of M3, in order to test the mediating effect of knowledge transfer on enterprise relationship network and open innovation behavior of SMEs. The results showed that, after adding the mediating variable, the explanatory variable relationship network passed the test at the significance level of 1%, but the regression coefficient decreased from 0.5295 to 0.1736. Based on the mediating effect test standard, it can be concluded that knowledge transfer partially mediates the relationship between SMEs’ participation in relationship networks and their open innovation behavior. This means that the positive impact of an enterprise’s relationship network on its open innovation behavior is partly achieved through knowledge transfer, as supported by hypothesis H5.

## 5. Discussion

Some research suggests that there is a complex relationship between the relationship network and the behaviors associated with open innovation [52–55]. Based on research to the research on the determinants of innovation in SMEs around two key drivers knowledge transfer [56],and innovation strategy orientation [57], this study analyzes what the mechanisms are by which knowledge transfer and innovation strategy orientation affect knowledge transfer, and relationship network affecting open innovation behavior of SMEs.

The results show that the relationship network positively affects open innovation behavior of SMEs. This finding aligns with some conclusions, which showed the significance of relationship network maintenance in this context [58]. The positive impact of the relationship network may result in enterprises reducing knowledge investments due to the potential for these investments to yield returns over time [59]. However, it is challenging for SMEs to sustain such investments, which require significant financial resources, thus hindering their capacity to make such investments [60]. Conversely, the findings of this study demonstrate a favorable influence of the enterprise relationship network on the open innovation behavior of SMEs, signifying an enhancement in the advantageous nature of prospective social relationships. This positive influence is particularly pertinent to decisions concerning innovation behavior.

The enterprise relationship network has been demonstrated to have a significant positive impact on knowledge transfer. Specifically, the results suggest that the relationship network can promote the efficiency of knowledge transfer. This finding indicates that the network effect has an indirect positive effect on innovation performance, while knowledge acquisition plays a partial intermediary role between the innovators’ network and their innovation performance [61].The relationship network provide incentives for knowledge transfer to engage in the utilization of knowledge, as it can be acquired at a relatively low cost in SMEs. Furthermore, the findings indicate that an increase in knowledge transfer is associated with an enhancement in open innovation behavior among SMEs. Because the SMEs don’t have the adequate knowledge accumulation to support innovation behavior all by themselves, in order to gain more knowledge, they prefer the strategy for open innovation behavior [62]. Additionally, knowledge transfer has been found to play a partial mediating role in the relationship between relationship network participation and open innovation behavior of SMEs, thereby indicating that the positive effect of an enterprise’s relationship network on its open innovation behavior of SMEs is partly realized through knowledge transfer.

The findings of this study underscore the pivotal role of relationship networks in driving open innovation among Chinese SMEs, mediated by knowledge transfer and moderated by innovation strategy orientation. These mechanisms operate within China’s unique collectivist cultural context, which fundamentally reshapes open innovation behaviors. The collectivist cultural framework in China, characterized by an emphasis on group harmony, shared goals, and collaborative networks, distinctively shapes SMEs’ open innovation behaviors compared to individualistic cultures [63]. Haier Group’s innovation ecosystem exemplifies this cultural influence [64]. Rooted in collectivist principles, Haier’s human-centered value co-creation model dismantles hierarchical structures into more than 4000 autonomous micro-enterprises that prioritize collective problem-solving over individual gains [65]. For instance, Haier’s HOPE platform—a collectivist-driven open innovation hub—facilitates cross-boundary knowledge transfer by aligning external innovators with internal small teams to co-develop solutions [66]. Haier’s collectivist orientation fosters relational embeddedness, its innovation strategy prioritizes ecosystem-wide value creation over firm-centric gains. When incubating IoT startups like Thunderobot (gaming PCs) and HYSTOU (medical robotics), Haier provided shared R&D infrastructure, supply chain access, and venture funds. Such outcomes highlight how collectivism amplifies relationship networks’ efficacy in open innovation: by institutionalizing trust and mutual obligation, Haier transforms transactional knowledge transfers into sustained co-innovation [67].

The results further demonstrate that the strength of innovation strategic orientation is directly proportional to the positive impact of relationship network on knowledge transfer. These results suggest that relationship networks increase available financial resources, thereby favoring knowledge transfer to SMEs. The results also support the argument that innovation strategic orientation has a greater capacity to permeate knowledge transfer when the decision to be made is complex and more knowledge is required to support it. The importance of relationship networks for SMEs is increased by innovation strategic orientation due to the orientation requiring more advanced knowledge to gain a competitive advantage for development [68].

### 5.1. Management implications

Firstly, it is imperative for enterprises to prioritize the cultivation of relationship networks in order to facilitate the open innovation behavior of SMEs in a cost-effective manner. A multitude of factors impede enterprises’ innovation activities. In the context of a market environment characterized by increasing uncertainty, enterprises are compelled to prioritize innovation that is both high-efficient and low-risk in nature. In the face of a complex and volatile market environment, the risk coefficient of enterprise operation and investment also increases accordingly, underscoring the necessity to strike a balance between efficiency and cost while formulating the overarching concept of open innovation behavior among SMEs. The creation of an open innovation concept that is both high-efficiency and low-risk within the enterprise is paramount in directing innovation efforts. Secondly, enterprises need to establish mechanisms to maintain and expand their relationships, keep abreast of the latest market information, and promote development through innovation. Finally, employees should be encouraged to communicate with other enterprises, through which they can learn from each other and contribute to the enterprise’s development, thus strengthening the stability of the enterprise’s relationships.

Secondly, in the contemporary business environment, enterprises must acknowledge the significance of knowledge transfer. In the current knowledge economy, enterprises must develop a profound understanding of the underlying motivations behind SMEs’ open innovation behavior. Innovation activities typically emerge during the process of knowledge transformation, and the open innovation behavior of SMEs is inextricably linked to the direct impact of knowledge transfer. Concurrently, enterprise management can prioritize enhancing employees’ knowledge levels by facilitating their participation in training activities both within and outside the enterprise. This approach ensures the internalization of transferred knowledge into the enterprise’s innovation activities, thereby fostering the emergence of open innovation behavior among SMEs.

Last, enterprises must have the consciousness of innovation strategic orientation. Firstly, enterprises must accurately identify and interpret the latest developments in the market and business sectors. They should then strive to identify and seize opportunities for acquiring new knowledge, thereby facilitating knowledge transfer and meeting innovation needs. Secondly, enterprises must establish a mechanism to adjust their strategic resources in accordance with market fluctuations, thereby facilitating the effective transfer of knowledge.

### 5.2. Limitations and future research directions

This paper proposed the hypothesis model based on theoretical analysis and made the survey data as reliable as possible, but there may still be limitations as follows: First, although the sample size is sufficient and there are a certain number of control variables, with the development and change of environments. Secondly, this paper explores the role of single mediating variable and single moderating variable on the main effect. Future research endeavors can delve deeper into the specific impact disparities of diverse types of relational networks (such as supply chain networks, industry alliances, and governmental partnerships) on the open innovation behavior of SMEs, as well as examining whether these networks exhibit differing mechanisms of action in the process of knowledge transfer. Furthermore, by employing a case study methodology and selecting representative SMEs for in-depth analysis, a more nuanced understanding of the dynamic interplay between relational networks, knowledge transfer, and open innovation behavior can be achieved. Additionally, incorporating a cross-cultural comparative perspective to analyze the similarities and differences in the influence of relational networks on SMEs’ open innovation behavior across different national and regional cultural contexts will broaden the applicability of existing theoretical frameworks. Moreover, leveraging big data analytics techniques to uncover the correlation patterns among relational network characteristics, knowledge flow patterns, and open innovation performance from vast enterprise data sets can provide robust empirical support for theoretical construction. Lastly, future research should also focus on the moderating role of environmental dynamism (such as the pace of technological change and shifts in market demand) in shaping the relationship between relational networks and open innovation behavior, thereby offering more refined guidance for SMEs to formulate effective innovation strategies in diverse market environments.

## 6. Conclusions

This study examines the interplay among enterprise relationship networks, knowledge transfer, and open innovation behavior within SMEs, with a particular focus on the moderating role of innovation strategic orientation. Empirical analysis reveals that corporate relationship networks significantly enhance knowledge transfer, an effect potentially amplified in collectivist cultures where trust-based reciprocal exchanges dominate. Furthermore, innovation strategic orientation not only directly promotes knowledge transfer but also strengthens the network-knowledge transfer nexus, suggesting that firms with pronounced innovation strategies derive greater value from relational capital. Both relationship networks and knowledge transfer exhibit robust positive impacts on SMEs’ open innovation behavior, with knowledge transfer partially mediating the network-innovation linkage. These findings underscore the strategic utility of relational embeddedness and cognitive adaptability in driving SME innovation ecosystems.

However, several limitations warrant attention. First, the sample’s geographic concentration in China may limit generalizability, as cultural and institutional contexts could influence network dynamics and innovation strategies. Second, reliance on cross-sectional survey data precludes causal inference regarding temporal sequences and dynamic adjustments. Third, while self-reported measures align with established scales, they may introduce social desirability biases, particularly in assessing strategic orientations.

Future research could address these gaps through multi-country comparisons to isolate contextual effects, employing longitudinal designs to trace evolutionary patterns, and integrating objective innovation metrics. Additionally, exploring contingent factors such as industry technological intensity or digital transformation maturity may refine theoretical boundaries. By advancing these dimensions, scholars can further elucidate the interplay between relational, cognitive, and behavioral mechanisms in SME innovation management.

## Supporting information

S1 Data Original Data1.
(XLSX)
